# GP at foot: remote ways to share management of diabetes amid the COVID-19 crisis

**DOI:** 10.3399/bjgp20X710201

**Published:** 2020-05-20

**Authors:** Ruth Chambers, Paul Beaney, Gayathri Balasubramanian, Seyi Ogunmekan

**Affiliations:** Digital Workstream, Staffordshire and Stoke-on-Trent Sustainability and Transformation Partnership (STP), Staffordshire.; Keele University, Keele.; Biomechanics and Rehabilitation Technologies, Faculty of Health, Staffordshire University, Stoke-on-Trent.; Furlong Medical Centre, Tunstall.

## BACKGROUND

The rising costs of managing diabetes account for 10% of the NHS budget, roughly 1 million GBP per hour, 80% of which is spent on treating complications of poorly controlled diabetes.^1^ GP practices are at the forefront of healthcare services helping people to manage their health conditions; especially now with the COVID-19 pandemic affecting frontline services. Therefore, personal interactive help from smart devices with digital assistants installed, such as Alexa, could greatly aid self-care. Feedback from 20 people with diabetes in our pilot project in Staffordshire indicates that such digital assistants could help to improve dietary choices and medication adherence now, and in the future, to manage footcare.

## CURRENT DIETARY ADVICE CONFUSION

According to the Staffordshire pilot project focus group participants, current diabetic dietary advice is *‘confusing’*, and *‘difficult to put into practice and maintain’*. These views are supported in the broader literature^2^^,^^3^ and on Diabetes UK support forums.^4^ It is easy to understand why. Most of us struggle to change our diets even for a short time, let alone a lifetime. Given that the NHS does not have the capacity to see patients more frequently and that patients should instigate their own individual dietary choices, solutions must support both perspectives.

## DIGITAL ASSISTANTS CAN HELP TO IMPROVE DIABETES MANAGEMENT

Participants reported that Alexa’s inbuilt functions were useful to improve their understanding of, and access to, dietary information, for example, *‘how many carbs in a slice of bread?’* Or *‘what chicken recipes do you know?’* One patient with type 1 diabetes found hearing how many carbohydrates were in a meal he was about to eat very useful for being able to calculate his required insulin, especially when he needed to eat quickly to avoid a hypo. Now he expects to be able to expand his dietary choices.

The ability to make lists, set reminders, and organise online calendars are inbuilt functions that are non-health specific but can be used for health purposes. The companion app (installed on a smartphone to access the device) ensured that even outside of their home patients were alerted to take their medications at the times they chose. Knock-on benefits were reductions in metformin doses by some, as well as self-reported improvements in their confidence that could be applied to self-care of other comorbidities.

## FUTURE POTENTIAL

Footcare was raised by participants as an area where they could see potential benefits of their new assistants. One of the problems shared was being unable to bend down to look under their feet as they had grown older. The device they trialled was voice activated, with a touchscreen and front-facing camera. Patients thought that these features would aid regular foot monitoring if they could be relayed via a dedicated diabetes footcare app for the device, with engaging ‘daily tips’ such as to check for wear and tear on shoes, and reminders about regular moisturising.^5^

Another suggestion was that their general practice or district nurses should have the free companion app so that the patient might be seen by videocall and their feet inspected remotely, generating a specialist review by the Foot Protection Service if justified;^6^ or even a ‘GP at foot’ video consultation review perhaps? Given strict ‘social distancing’ measures and reduced NHS capacity, solutions like these could be very helpful ways for shared management of patients’ long-term health conditions.

## KEY DEVELOPMENT AREAS

However, there is room for improvement with such digital assistants. For example, our participants discovered that some of the health advice, recipes, and nutritional information given often originates from US sources, while specific diabetes information was limited and not from a trusted UK source, thus potentially creating unhelpful confusion. Safeguards over patient data and use of these devices also need to be incorporated if video consultations were introduced.

To summarise, for people with diabetes, a digital assistant in the home can provide access to key nutritional information and boost medication, diet, and footcare regimes. With further development and integration, this technology has the potential to reduce complications that often arise insidiously with life-changing consequences. With diabetes care costs spiralling and NHS resources being overstretched, we must take up the opportunities that intelligent digital assistants will give our patients in the current acute healthcare crisis and ongoing chronic healthcare demands.
